# The Induced Membrane Technique—The Filling Matters: Evaluation of Different Forms of Membrane Filling with and without Bone Marrow Mononuclear Cells (BMC) in Large Femoral Bone Defects in Rats

**DOI:** 10.3390/biomedicines10030642

**Published:** 2022-03-10

**Authors:** René D. Verboket, Nicolas Söhling, Myriam Heilani, Charlotte Fremdling, Alexander Schaible, Katrin Schröder, Jan C. Brune, Ingo Marzi, Dirk Henrich

**Affiliations:** 1 Department of Trauma, Hand and Reconstructive Surgery, Goethe University Frankfurt, 60590 Frankfurt am Main, Germany; nicolas.soehling@kgu.de (N.S.); myriam.heilani@hotmail.com (M.H.); charlotte.fremdling@gmx.de (C.F.); alexanderschaiblex@gmail.com (A.S.); marzi@trauma.uni-frankfurt.de (I.M.); d.henrich@trauma.uni-frankfurt.de (D.H.); 2 Center of Physiology, Cardiovascular Physiology, Goethe University Frankfurt, 60590 Frankfurt am Main, Germany; schroeder@vrc.uni-frankfurt.de; 3 German Institute for Cell and Tissue Replacement (DIZG, gemeinnützige GmbH), 12555 Berlin, Germany; j_brune@dizg.de

**Keywords:** critical size defect, tissue engineering, BMNC, Masquelet technique, bone regeneration

## Abstract

The Masquelet technique is used to treat large bone defects; it is a two-stage procedure based on an induced membrane. To improve the induced membrane process, demineralized bone matrix in granular (GDBM) and fibrous form (f-DBM) was tested with and without bone marrow mononuclear cells (BMC) as filling of the membrane against the gold standard filling with syngeneic cancellous bone (SCB). A total of 65 male Sprague–Dawley rats obtained a 5 mm femoral defect. These defects were treated with the induced membrane technique and filled with SCB, GDBM, or f-DBM, with or without BMC. After a healing period of eight weeks, the femurs were harvested and submitted for histological, radiological, and biomechanical analyses. The fracture load in the defect zone was lower compared to SCB in all groups. However, histological analysis showed comparable new bone formation, bone mineral density, and cartilage proportions and vascularization. The results suggest that f-DBM in combination with BMC and the induced membrane technique cannot reproduce the very good results of this material in large, non-membrane coated bone defects, nevertheless it supports the maturation of new bone tissue locally. It can be concluded that BMC should be applied in lower doses and inflammatory cells should be removed from the cell preparation before implantation.

## 1. Introduction

Bone defects of critical size due to tumor resections, osteomyelitis, or trauma are a huge challenge for surgeons and patients [1]. The critical size in a bone defect is defined as either a bone loss greater than “50% of the circumference of the particular bone” [2] or a defect in a bone that “will not heal during the lifetime […] or a defect that shows less than 10% regeneration” [2]. To treat these defects correctly, different surgical and biomedical approaches are available. Autologous bone grafting, bone transport techniques (distraction osteogenesis) or vascularized free bone transfer [3,4] are some of the methods used, yet they are afflicted with several limitations in use. Restricted bone volume at the donor site and donor site morbidity such as long-lasting pain are described [5]. To avoid these limitations, various procedures have been developed. One of the most commonly used clinical procedures is the induced membrane technique developed by Masquelet et al. [6]. In an initial operation, a bone cement (PMMA) spacer is inserted into the defect. After 6–8 weeks, a membrane has formed around the spacer. After removal of the spacer in a second surgical step, the membrane can be filled with autologous bone or scaffolds [7,8,9,10]. The function of the membrane was described by Masquelet (2010). The membrane serves as a “bioreactor” that provides a barrier against the surrounding soft tissue [7]. Manipulating the induced membrane to further enhance bone healing is currently considered a key technique to improve bone healing. In our group, it has been shown that different additives to the bone cement have an influence on the membrane formation [11].

Various types of cells and biotechnological processes have recently been used to accelerate the healing of defects in bone and soft tissue. Stromal vascular fraction (SVF) and the decellularized extracellular matrices (ECM) were used in tissue regeneration [12,13,14]. Additionally, different platelet-rich plasma (PRP) combinations were used to support regenerative processes [12,15,16,17,18,19,20,21,22,23].

The cellular composition and the amount of growth factors in the membrane also have a decisive influence on the function even over a longer period of time [24,25]. Several research groups have been able to show that the harvest of autologous bone, and the previously mentioned disadvantages, can be reduced by the use of synthetic scaffolds such as tricalcium phosphate, hydroxyapatite or demineralized bone matrix to fill the defect. Relatively good results were achieved in combination with osteoinductive cells such as bone marrow-derived mononuclear cells (BMC) [11,26,27,28,29,30,31,32,33]. The scaffolds analyzed in the previous studies exerted various effects on bone healing due to the different surface to volume ratios [34,35,36,37,38]. Due to variable particle sizes stability can be achieved. A denser insertion of scaffolds into the defect can influence the sprouting of blood vessels and thus the supply of nutrients [38].

In our own preliminary work using a 5 mm femoral defect in the rat, it was shown that fibrous demineralized bone matrix (f-DBM) is toxicologically safe and significantly supports bone defect healing in two thirds of cases [39]. In another study, the same model was used to demonstrate that f-DBM seeded with BMC also supported bone defect healing, but it was partially inferior to the vital syngeneic bone graft in parameters such as biomechanical loading capacity and mineralization [40].

In another previous work, it was shown that BMC in combination with the induced membrane technique and a mineral scaffold led to a further improvement of various bone healing parameters compared to the parallel approach without induced membrane [28].

Therefore, to combine the advantages of the above treatment approaches, the effect of BMC-colonized fibrous demineralized bone matrix or granular demineralized bone matrix in combination with the induced membrane technique was investigated using a 5-mm femoral defect of the Sprague–Dawley (SD) rat compared with the uncolonized test materials and syngeneic bone material after 8 weeks of healing.

## 2. Materials and Methods

### 2.1. Ethics

All animal experiments were performed according to the applicable regulations of the Animal Protection and Monitoring Committee of our institution (project no. FK/1075; Regierungspräsidium, Darmstadt, Germany) in accordance with German law.

### 2.2. Animal Care and Group Setup

A total of 65 8- to 10-week-old male Sprague–Dawley rats (Janvier Labs, Saint Berthevin, France) with a weight of approximately 250–300 g were housed with 3 to 4 animals per cage. The housing conditions were equal for all rats: temperature (15–21 °C), air circulation, and light-controlled rooms (14 h day and 10 h night). The rats had unlimited access to rat food and water. Veterinarians monitored the animals’ well-being daily for the first week after surgery and weekly thereafter.

**Figure 1:**
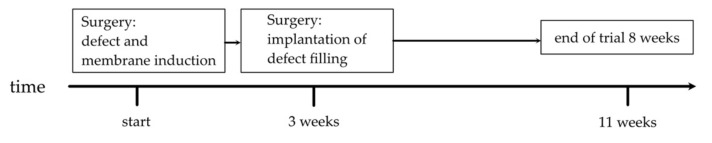
Procedure of the animal tests.

The animals were randomly divided into five test groups and underwent two operation steps (Figure 1). Each group received different DBM preparations, supplemented in some groups with syngeneic BMC isolated from donor rats, and a control group in which bone defects were filled exclusively with syngeneic cancellous bone (SCB) isolated from donor animals without the addition of BMC. The latter filling corresponds to the current clinical standard of care. The femoral defects of the animals in the second group were filled with granular demineralized bone matrix (GDBM). In the third group, the same material as in the second group was used but with additional loading of BMC. Fibrous DBM (f-DBM) was used in the fourth group and f-DBM loaded with BMC in the fifth group. The DBM granules used (Deutsches Institut für Zell-und Gewebeersatz (DIZG) gemeinnützige GmbH, Berlin, Germany) were sterilized according to a validated GMP-compliant procedure and they were approved as a medical product according to §21 of the German Drug Law (approval number: PEI.H.03358.01.1). Fibrous DBM is manufactured under controlled computerized numerical control (CNC) milling conditions. The tissue is then partially demineralized and subjected to a validated sterilization process [41]. All DBM products are manufactured from bones of serologically tested donors. The manufacturing processes are fully validated, including decellularization, sterilization, and preservation [41] (Table 1).

### 2.3. BMC Isolation and Seeding

Syngenic rats served as donors for BMC. The femora and the tibiae were removed under sterile conditions and the condyles were cut using a sterile side cutter. The bone marrow was rinsed from the medullary cavity using a 5 mL sterile syringe with PBS mixed with 1% antibiotics and suspended by pipetting several times. After dilution of the bone marrow with PBS (1:3), bone marrow mononuclear cells were isolated by Ficoll density gradient centrifugation (1.077 g/cm^3^, Biochrom, Berlin, Germany) at 800 *g* for 20 min without brake at 20 °C. After two washes with 25 mL PBS each (800 *g*, 7 min), BMCs were counted and adjusted to a concentration of 1 × 10^7^ cells/mL for further use. A FACS analysis (FACScalibur, BD Biosciences, Heidelberg, Germany) was performed to verify the quality of BMC preparation. The BMCs were identified based on their forward scatter and side scatter characteristics [42].

**Figure 2:**
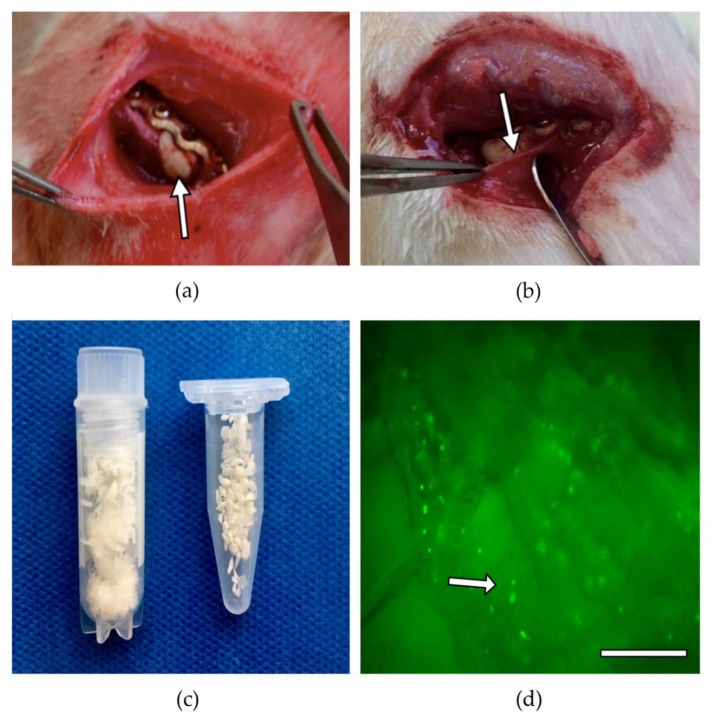
Plate stabilized femoral defect with cement spacer (white arrow) to induce membrane (**a**). A 5-mm critical size defect in the right femur of a rat is stabilized with a 5-hole-plate, after 4 weeks the induced membrane (white arrow) is visible. Macroscopic image of the membrane after removal of cement spacer (**b**). Macroscopic image of f-DBM left and GDBM right, same amount (weight) is shown (**c**). Fluorescence microscopic image, adherent CFSE-stained BMC (green dots, marked with white arrow) can be seen at different depth levels of the f-DBM (original magnification 50×) (**d**).

The cell seeding was done as previously described [40]. Using autoclaved forceps, the scaffolds, which were weighed and delivered pre-portioned by the manufacturer, were placed in individual wells (area 2 cm^2^) of a 24-well plate (Nunc, Wiesbaden, Germany). In each case, 100 µL of a cell suspension containing 28.2 × 10^5^ BMC [43] was carefully applied dropwise to the scaffolds. After a 5-min incubation at 37 °C, the cell suspension that was not absorbed by the scaffold material was pipetted onto the material again. After another incubation (5 min, 37 °C), the cell loading procedure was completed. Evidence that BMC adhered to the scaffold was obtained in a simultaneous experiment using 5(6)-carboxyfluorescein 3’,6’-diacetate N-succinimidyl ester (CFSE) pre-stained BMC. The BMCs adhering to the material were visualized by fluorescence microscopy (Figure 2). Staining of the BMC with CFSE was performed according to the manufacturer’s protocol (Thermo Fisher, Waltham, MS, USA). In brief, 5 × 10^6^ BMC were incubated in 10 mL of a 1 µM CFSE solution prewarmed to 37 °C for 20 min at 37 °C in a water bath. After centrifugation (400× *g*, 10 min), the BMCs were resuspended in DMEM + 10% FCS and incubated for an additional 30 min at 37 °C. For seeding, CFSE-stained BMCs were centrifuged and resuspended in PBS at a density of 28.2 × 10^6^ BMC/mL.

### 2.4. Surgical Procedure

The surgical procedure was performed as previously described [40]. The animals were anaesthetized via an intraperitoneal injection of Ketavet and Rompun. All subsequent procedures were performed under sterile conditions. For the creation and the internal fixation of the bone defect, the right femur was carefully exposed surgically. The internal fixator, a 5-hole plate, (Miniplate Lockingplate LCP Compact Hand 1.5 straight, DePuy-Synthes, Dubendorf, Switzerland) was placed in an adequate position on the femur and fixed with four 1.3 mm angular stable cortical screws (Compact Hand, DePuy-Synthes). The central hole was left free. A 5 mm osteotomy was created below the central hole using a Gigli saw (RI-Systems, Landquart, Switzerland), and PMMA cement (Palacos, Heraeus Medical, Wehrheim, Germany) that was freshly prepared according to the manufacturer’s instructions, was filled into the bone defect (Figure 2). Previous studies showed that a 5 mm defect represents a critical defect [43]. To cool the exothermically setting PMMA, the defect area was rinsed with physiological NaCl solution. The wound was closed in two layers with continuous subcutaneous stiches using a 4/0-monofilament nylon suture and the animals were placed in their cages. The animals were examined daily for complications and behavioral changes. Analgesia (2.6 mg/kg Carprofen s.c. directly after surgery) was administered postoperatively and via the drinking water (Tramadol, 2.5 mg/100 mL) for 7 postoperative days.

After three to four weeks, the second operation was performed. The induced membrane that formed around the PMMA spacer was opened and the spacer was removed. After refreshing the fracture ends, the membrane pocket was filled with scaffold variants or syngeneic cancellous bone according to the experimental and randomization protocol. Wound care and pain management were the same as in the first operation. The rats were kept under standard conditions and standard diet for eight weeks until the final bone harvest. The animals were euthanized under inhalation anesthesia and intracardiac pentobarbital injection (500 mg/kg body weight).

The femora were stored at −80 °C after removal of the plate. The analyses were performed on the specimens in the following sequence: µCT analysis, biomechanical analysis, and (immuno)histological analysis. This allowed the sample material to be used optimally and the number of animals needed to be significantly reduced [40].

### 2.5. Biomechanical Characterisation

For preservation, the bones were stored in 70% ethanol until the start of the biomechanical analysis. Using a material testing machine (Zwicki-Line Z5.0, Zwick-Roell, Ulm, Germany), the mechanical properties of the bone defect zone were measured via a three-point bending test. Loading and bending of the specimen were recorded continuously. The Testexpert-II software (Zwick-Roell, Ulm, Germany) was used to record and evaluate the measured data. Bending stiffness and ultimate load were evaluated. The ultimate load was defined as a rapid force drop of at least 50%; the bending stiffness, in accordance with the literature, ‘was calculated from the slope of the linear elastic portion of the (load-deformation) curve’ [44]. In the next step, the measured values were set in relation to the respective contralateral, healthy femur. After biomechanical testing, the femora were stored in 70% ethanol.

### 2.6. Histological Assessment

The formation and the maturation of new bone tissue in the defect area was analyzed histomorphometrically on the basis of Movat pentachrome-stained, decalcified histological section preparations, as previously described in [30]. The bone preparations were fixed in Zinc-Formal-Fixx (10%, Thermo Electron, Pittsburgh, PA, USA) for 20 h. Subsequently, the femora were decalcified over a period of 14 days in an aqueous solution containing 10% EDTA (Sigma-Aldrich, Taufkirchen, Germany) and 0.25 M Trizma base (Sigma-Aldrich, Taufkirchen, Germany). The pH of the solution was set to 7.4. After embedding the decalcified bones in paraffin, longitudinal sectional preparations (3 µm thickness) were made. Movat pentachrome staining was performed according to Garvey et al. [45] using a staining kit (Morphisto, Frankfurt, Germany) and following the manufacturer’s instructions. The percentage of bone tissue and cartilage tissue was calculated in relation to the defect area using ImageJ software (V 1.53g, U. S. National Institutes of Health, Bethesda, MD, USA). For osteocalcin staining, sections were incubated with monoclonal mouse anti-rat osteocalcin antibody (Abcam, Cambridge, UK, ab13418, clone OC4-30, lot GR308720-4) or for a-SMA staining with polyclonal rabbit anti-α-SMA (Abcam, ab5694, lot GR3356867-3) for 1 h at room temperature at final concentrations of 2 μg/mL each. To visualize the antigens, the sections were then incubated for 30 min with the secondary antibody, a polyclonal HRP-conjugated anti-mouse or anti-rabbit IgG (Simple Stain Rat MAX PO, Nichirei, Tokyo, Japan), which was followed by a 3-amino-9-ethylcarbazole (AEC, Sigma-Aldrich, Taufkirchen, Germany) incubation. Then, counterstaining with hematoxilin was performed. To prevent misinterpretation of α-SMA positive cells as small blood vessels, only α-SMA positive areas larger than 10 µm were included in the analysis. The histological preparations were analyzed by light microscopy. High-resolution panoramic images of the entire defect area were obtained by automatically combining individual images using the BZ-9000 microscope (Keyence, Neu-Isenburg, Germany) and the BZII Analyzer Software (Keyence, Neu-Isenburg, Germany). The images were analyzed by two independent examiners who were blinded to group assignment. A bone healing score was used to assess the extent of bone defect healing [46]. Improved bone healing is characterized by higher values.

### 2.7. μCT-Analysis

A µCT analysis was performed to determine bone mineral density and callus volume using a high-resolution in vivo micro-CT Skyscan 1176 (Bruker AXS, Karlsruhe, Germany). The long axis of the femur was lined up orthogonally to the axis of the X-ray beam (Al 0.5 mm; voltage: 50 kV; current: 500 μA; frame average: 7; rotation ra.: 180; rotation s.t.: 0.5) and the region of interest was placed on the defect. The isotropic voxel size was 18 μm3. Two-dimensional CT-images were scanned, reconstructed using a standard back convolution procedure, and saved in 3D arrays. After µCT-scanning, the femora were stored in 70% ethanol.

To determine the bone mineral density in the defect area, a phantom with known density was measured in parallel. For the calculation of the total volume and the bone volume, a global threshold (60 to 240 grey levels) was defined in the relevant defect volume. For this purpose, a virtual cylinder 5 mm in length was placed centrally in the middle of the defect and the values for total volume and bone volume were calculated using ImageJ (https://imagej.nih.gov/ij/notes.html, accessed on 24 October 2021). Due to the high radiological similarity between syngeneic, grafted bone and newly formed, already calcified bone tissue, as well as between DBM and not yet fully mineralized bone tissue, newly formed bone tissue could not be reliably detected, comparable to previous studies [36]. Therefore, the total volume and the bone volume of the defect were determined.

### 2.8. Statistics

The results of the experiments performed are presented as box plots (median) in the figures or as mean and standard deviation in the texts. To compare the groups, a nonparametric Kruskal–Wallis test with Bonferroni–Holm-corrected Conover–Iman post-hoc analysis was used with the statistical software Bias 11.10 (Epsilon-Verlag, Darmstadt, Germany). The *p* values < 0.05 indicate statistical significance.

## 3. Results

### 3.1. Animal Care/Complications

A total of five animals were excluded from the evaluation. Three animals were excluded due to loosening of a screw, and two animals died due to respiratory failure during surgery. The grafts were considered safe for use in previous experiments. There were no macroscopic side effects [39].

### 3.2. Similar Biomechanical Properties in All Induced Membrane Groups

**Figure 3:**
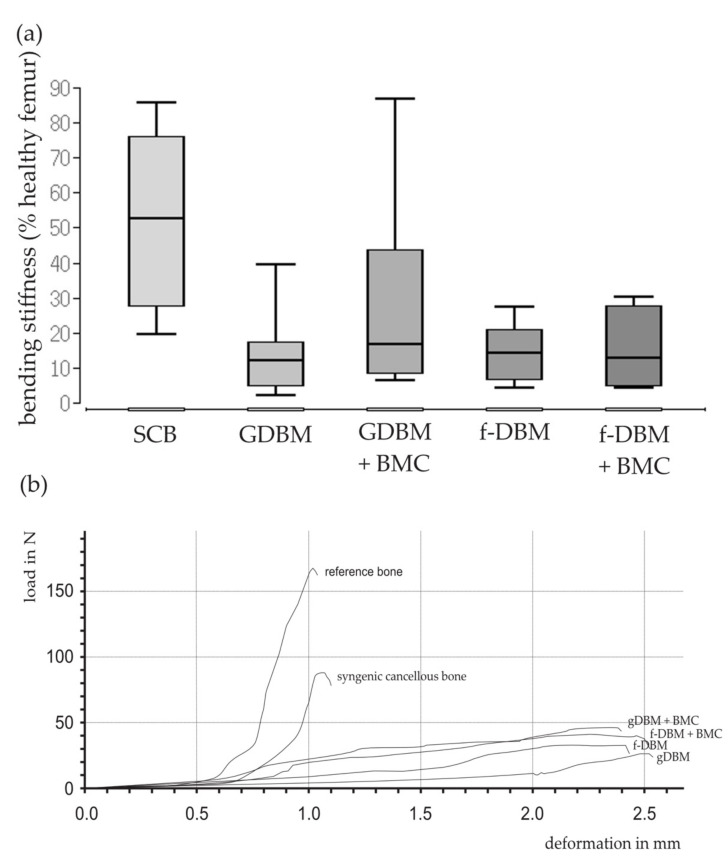
Bending stiffness of the 5 mm bone defect in relation to the contralateral healthy femur (=100%). The percentage of bending stiffness as a percentage of the healthy contralateral femur was lower in all induced membrane groups. No significant differences between the cell and the non-cell groups (**a**). Selected force/deformation curves of the different groups show the deformation in mm per load in newton (**b**).

The ultimate load as percentage of the healthy contralateral femur was observed to be lower in all membrane groups than in the SCB group. No significant differences between the cell and the non-cell groups and the DBM or the f-DBM groups were detected. The highest median of all groups was observed in the SCB group (median: 52.8%) (Figure 3).

### 3.3. Significantly Better Bone Formation in GDBM + BMC vs. fDBM Group and Similar Cartilage Formation in All Groups

The analysis of the histological slides stained with Movat pentachrome revealed significantly better bone formation in the GDBM + BMC vs. the fDBM group (median: 63.2% vs. fDBM *p* < 0.05) and no significant differences in bone or cartilage formation between the other treatment groups.

**Figure 4:**
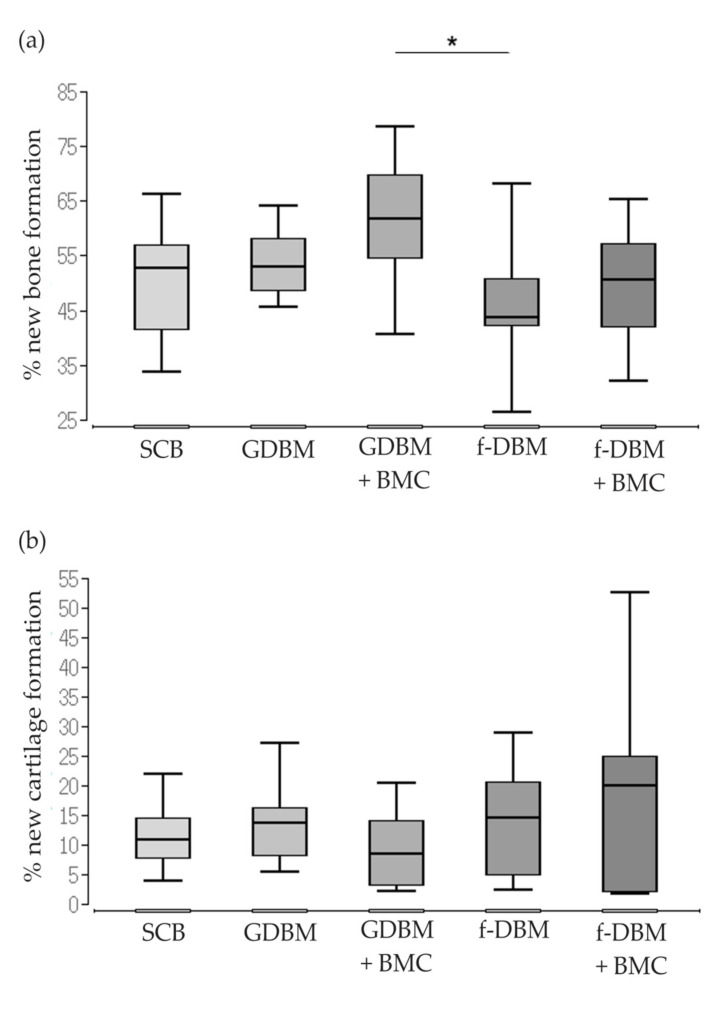

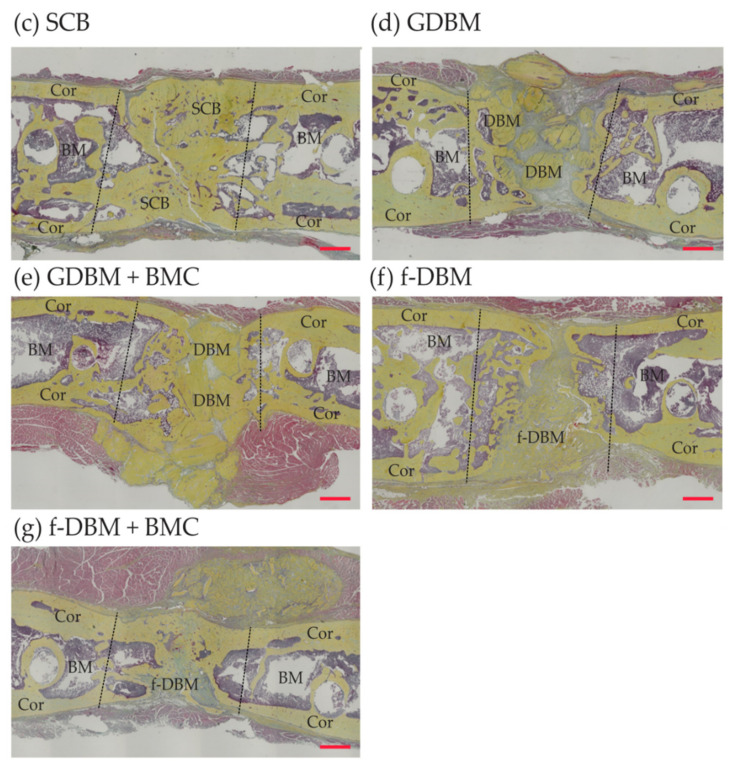
Percentage of new bone formation (**a**) and percentage of new cartilage formation (**b**). Significantly higher % of new bone formation in GDBM + BMC vs. f-DBM group (* = *p* < 0.05). Movat-pentachrome staining of SCB (**c**), GDBM (**d**), GDBM + BMC (**e**), f-DBM (**f**) and f-DBM + BMC (**g**). Bone tissue appears yellow, cartilage greenish. Histology: Size Bar represents 1 mm, dotted line defect zone, BM = bone marrow, Cor = corticalis.

The extent of new bone formation in defects filled with GDBM + BMC was most prominent (median value: 63.2%). Comparably high values were recorded in the two fiber groups (median f-DBM: 44.8%; median f-DBM + BMC: 52.7%), but the differences between the groups were without statistical significance. Moreover, the highest cartilage formation was measured in bone defects filled with f-DBM + BMC (median: 20.6%) (Figure 4).

### 3.4. Highest Bone Healing Scores in Syngenic Cancellous Bone and f-DBM Groups

Overall, the bone healing score values were at a similar level between the groups, therefore existing differences could not be statistically validated. The highest score values were obtained using defect filling SCB with 22 points, followed by the fDBM group (20 points). The lowest score of 18 points was observed using defect fill GDBM (Table 2).

### 3.5. No Significant Differences Bone Mineral Density

**Figure 5:**
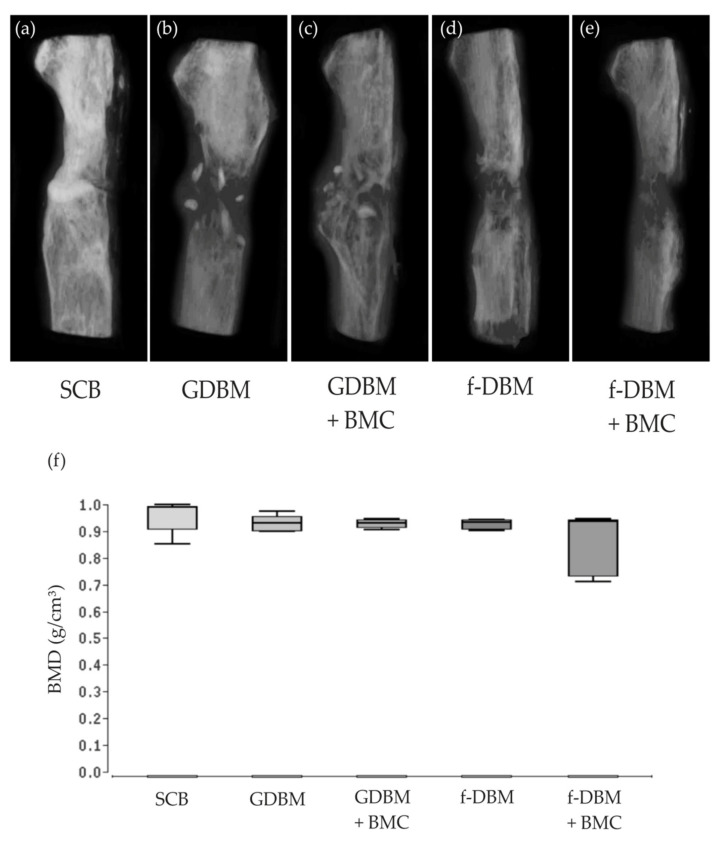
Radiological analysis of bone healing in the different groups, 3D µCT reconstructions are shown in (**a**–**e**). (**a**): SCB with high degree of osseous integration. (**b**): GDBM with low bone forming activity and few remaining implant granules. (**c**): Additional BMC led to a higher degree of integration and fewer remaining granules. (**d**): f-DBM demonstrates a high degree of osseous integration and almost complete bony bridging. (**e**): f-DBM + BMC led to evenly callus formation but no complete bridging. Bone mineral density (BMD) in the defect area in the experimental groups. (**f**): No significant differences were detected between the groups.

The µCT examinations (Figure 5a–e) showed respectable bone remodeling and bone formation in all groups; complete osseous defect bridging was observed in the SCB group (Figure 5a). The GDBM group showed a considerable amount of the initial defect size and the few remaining DBM granules. In the GDBM + BMC group, a higher degree of osseous integration of the implanted material and fewer remaining granules was noted. The f-DBM group showed a good osseous integration of the implant and almost complete bony bridging. In the f-DBM + BMC group, even callus formation but no complete bridging was frequently observed (Figure 5f). Bone volume to total volume ratio (BV/TV) was also measured in the µCT scans. The highest BV/TV was measured in the SCB group 0.78 (±4.4%), followed by GDBM + BMC 0.73 (±5.1%). The lowest value was detected in the GDBM group 0.61 (±4.2%). No significant differences were detected (Table 3).

### 3.6. Significantly Improved Bone Maturation in f-DBM + BMC vs. SCB, vs. GDBM, and vs. GDBM + BMC—No Differences in Vascularization

**Figure 6:**
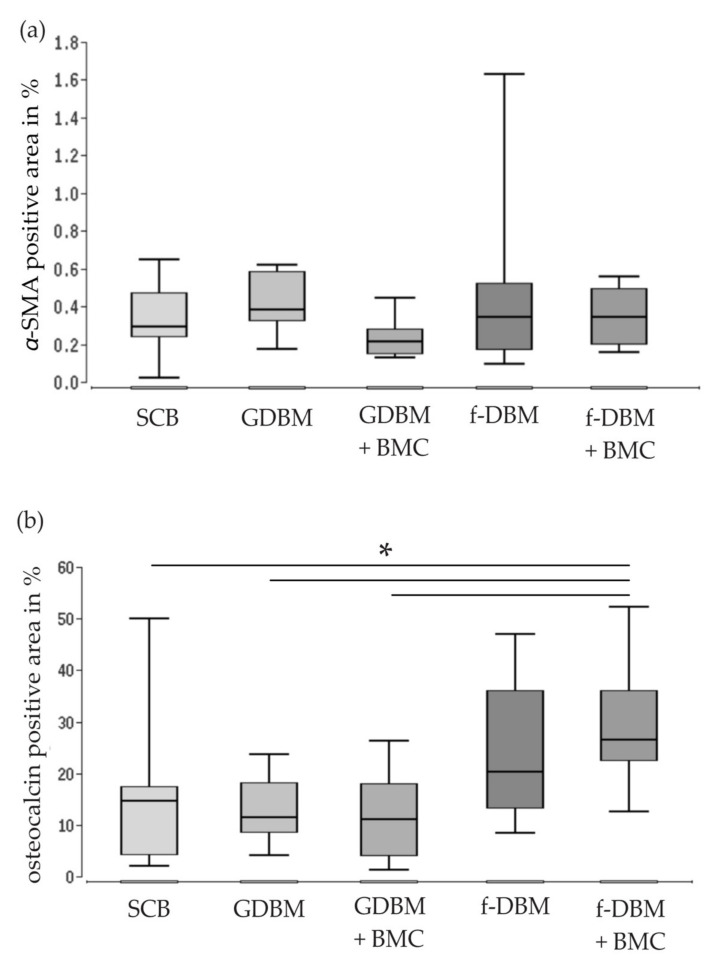

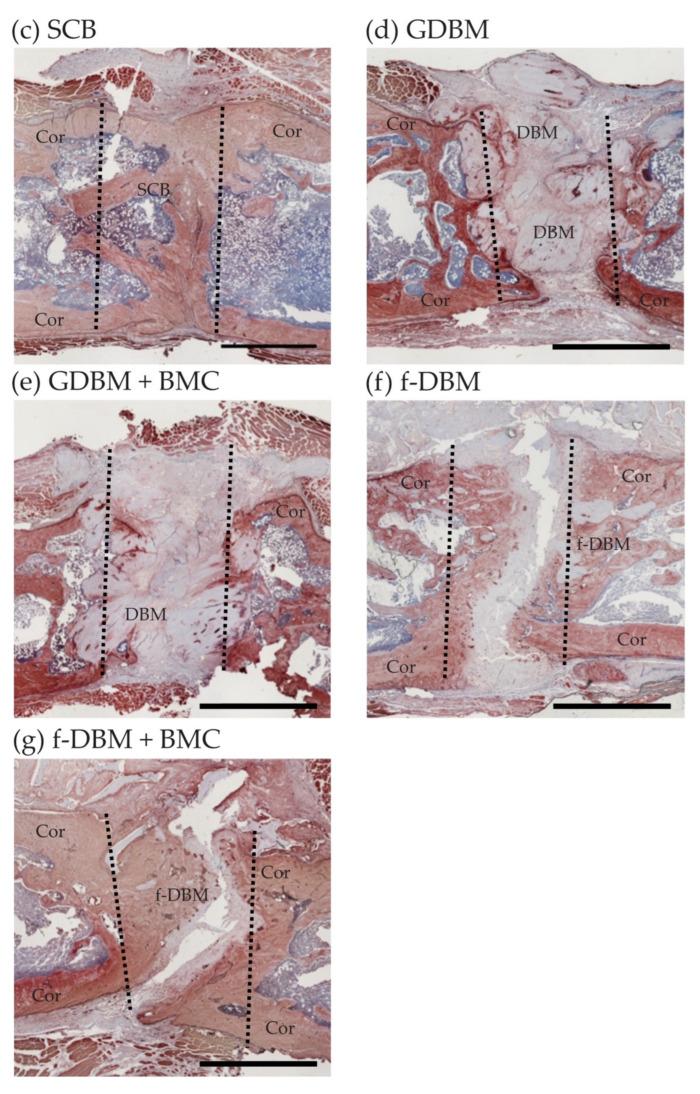
Histological determination of α-SMA-positive vessels (**a**) and osteocalcin expression (**b**–**g**) within the defect area. Uniform degree of vascularization was observed in all treatment groups. Evaluation of vascularization based on immunostaining of α-SMA positive, established blood vessels within the whole defect. Significantly higher osteocalcin expression in bone defects treated with f-DBM + BMC vs. SCB, vs. GDBM, and vs. GDBM + BMC (* = *p* < 0.05). Representative images of the osteocalcin staining (**c**–**g**). Osteocalcin-positive areas appear brownish. Bars represent 5 mm, dotted line defect zone, Cor = corticalis.

No significant differences in vascularization were detected via α-SMA staining. The largest α-SMA positive area was found in the GDBM group (median: 0.37%). The addition of BMC did not lead to a further increase of the α-SMA positive area (Figure 6a). Bone maturation in the defect area was assessed by osteocalcin staining. The osteocalcin positive area was significantly increased in f-DBM + BMC- vs. SCB-, vs. GDBM- and vs. GDBM + BMC groups (* = *p* < 0.05). Most osteocalcin positive areas were detected in the f-DBM + BMC group (median: 27.8%), with the lowest values in the GDBM + BMC group (median: 11.2%) (Figure 6b).

## 4. Discussion

In this animal experimental study, the effect of fibrous BMC-populated demineralized bone matrix (f-DBM) in combination with the induced membrane technique (Masquelet technique) on bone defect healing after 8 weeks of healing time using the 5 mm femoral defect of the SD rat was analyzed in comparison to DBM granules and syngeneic cancellous bone.

It was found that the fracture load in the defect zone was lower by a factor of three compared to the syngeneic cancellous bone in all experimental groups, but this was not statistically significant. However, histological analysis showed comparable new bone formation, bone mineral density, and cartilage proportions and vascularization in all groups compared with the cancellous bone group. Interestingly, however, osteocalcin expression was significantly increased in the f-DBM + BMC group compared with the SCB and the GDBM +/− BMC groups.

The induced membrane technique is a two-stage procedure that promotes the healing of large bone defects using cancellous bone material as a defect filler [7]. This general finding was confirmed in this study, with the biomechanical loading capacity in the corresponding experimental group reaching a median of more than 50% of the values of healthy bone. Without an induced membrane, this value is significantly lower at 20–30% (median) as shown by our research group using the same defect model in previous studies [47,48]. If a bone substitute material is used instead of cancellous bone, only a very weak biomechanical strength is usually given without an induced membrane, but this can be significantly increased by the addition of regenerative cells such as MSC, EPC, or BMC [29,42,49,50] and further raised in the context of the induced membrane technique [28]. This was observed in a previous study using a mineral scaffold (β-TCP) in combination with MSC and EPC or BMC. However, levels of syngeneic cancellous bone were not achieved in combination with induced membrane and scaffold supplemented with regenerative cells [28].

In the study presented here, the addition of BMC does not result in any further increase in the biomechanical loading capacity of the bone defect zone, but the radiological and histological images show an increase in bone tissue and osseous development of the defect, at least when granular DBM and BMC are used but not when fibrous DBM is used.

The increase in ossification activity when granular DBM is used is likely an effect of BMC. Similar observations have been made in a similar animal model. For example, Janko et al. [27] showed comparable results using a 5 mm femoral defect in the athymic nude rat, albeit without an induced membrane. Analogous to the study presented here, bony fusions of the BMC-colonized DBM granules with the fracture ends as well as with each other could also be observed there, which partially led to a complete osseous bridging of the defect compared to non-colonized DBM.

In contrast to granular DBM, fibrous DBM with the addition of BMC showed a tendency to less new bone formation radiologically and histologically, when compared to f-DBM without BMC and GDBM. In a previous study, however, complete bone healing was observed in one third of the cases and at least significantly advanced bone healing activity in another third of the cases when using f-DBM, also in a 5 mm defect in the rat femur [39]. In the aforementioned study, however, no induced membrane technique was used, and no cell preparation was added. The restoration of a bone defect with f-DBM and BMC without an additional membrane envelope showed good healing results in terms of biomechanical properties and the extent of new bone formation in another study using the 5 mm femoral defect [40]. Therefore, for f-DBM, the specific use case seems to be of greater importance. The results of the present study and previous analyses [39,40] suggest that f-DBM may lose effectiveness in the context of membrane techniques. At present, we can only speculate about the reasons for this. It is conceivable that due to the large surface/volume ratio and additionally introduced BMC the cell density in the defect is probably higher compared to the use of GDBM. Our own preliminary work and studies by other research groups show that high cell densities in the bone defect can be counterproductive for bone defect healing. For example, Janko et al. [51], in a dose–response experiment using the 5 mm femoral defect model and a mineral scaffold, showed that a tenfold higher cell dosage compared to that chosen here leads to a deterioration of various bone healing parameters. In another study, also using athymic nude rats, 1*10^7^ mononuclear cells (containing approximately 1 × 10^5^ CD34+ hematopoietic stem cells) on a collagen-based scaffold were implanted into a 2 mm femoral defect. In this group, a significantly higher non-union rate and a significantly stronger expression of proinflammatory mediators were observed compared to animals receiving only 1 × 10^5^ CD34+ hematopoietic stem cells [52]. These results suggest that an excess of mononuclear cells in the defect area significantly worsens new bone formation and thus bone defect healing via increased inflammation. Provided that GDBM binds significantly less BMC than f-DBM and thus the cell concentration in the defect is lower, this hypothesis finds confirmation in our results. When GDBM supplemented with BMC was used, a trend toward improved new bone formation was observed histologically and radiologically compared with defects filled with BMC-loaded f-DBM.

An additional factor in this scenario is the barrier effect of the induced membrane. In our preliminary work, histological analyses showed vascularization of the membrane surrounding the defect but no direct connectivity of the blood vessels with the defect area, whereas without the membrane the defect filling is surrounded by well-perfused muscle tissue, which could potentially allow a better supply of the defect zone [47,53]. It is conceivable that an insulating effect of the membrane may prolong the retention time of proinflammatory mediators in the defect area, thus additionally amplifying and perpetuating an adverse inflammatory response. Furthermore, it is conceivable that there is an increased nutrient and oxygen demand in the defect area due to implanted cells, but this demand cannot be met because of initial inadequate vascularization. This could lead to an increased rate of cell death and a consequent release of damage-associated molecular pattern (DAMP) from necrotic cells [54]. Released DAMPs may result in further activation of the inflammatory processes, thus contributing to a deterioration of bone healing [55]. However, the exact processes are currently purely speculative and they require further analysis.

These assumptions are at least partially consistent with the results of the BMD analysis. Even though no significant differences between the treatment groups and the control group could be found, there was a tendency for lower values in defects colonized with f-DBM filled with BMC, which could also indicate an impaired bone healing process in this group.

Somewhat contradictory to this are the results of osteocalcin expression. Here, the highest values were recorded in the f-DBM groups. This could indeed be due to an improved maturation of the newly formed bone tissue, which could be supported due to the osteogenic factors contained in the DBM, such as BMP2 [56,57]. In addition, due to the large surface/volume ratio of the f-DBM, these factors might be more accessible locally but perhaps only over a shorter period of time.

Interestingly, immunohistochemically determined vascularization density did not benefit from the addition of BMC and it was also independent of the type of DBM used. Previous analyses by our group demonstrated that BMC supports vascularization activity primarily in the earlier phases after bone defect creation. Thus, significantly increased vessel densities could be demonstrated 14 days after defect creation using GDBM as a defect filler [27]. Then, in a further study that is consistent with the results presented here, using BMC seeded GDBM and f-DBM without a membrane envelope, no significant differences in blood vessel density were recorded after 8 weeks of healing [40]. In another analysis using the induced membrane technique but applying a mineral bone graft substitute, there were also no differences in vascularization between scaffolds with or without BMC colonization after 8 weeks of healing [53].

As imitations of this work, we see the transferability of a rat model to humans, which is always given in animal experiments. In addition, further studies are needed to evaluate exact mechanistic aspects, especially in the early phase of bone healing. In the µCT examination, it is difficult to distinguish newly formed bone from introduced foreign bone-based material so a histological evaluation must always be performed.

## 5. Conclusions

In summary, the study presented here shows a rather heterogeneous picture of the efficacy of f-DBM for bone defect healing if used as a defect filler in the context of the induced membrane technique. While possibly supporting maturation of new bone tissue locally, the majority of results rather suggest that f-DBM in combination with BMC and the induced membrane technique cannot reproduce the very good results of this material in large, non-membrane coated bone defects. Thus, it can be concluded that cell therapeutics should be applied in lower doses and that additional possibly inflammatory cells should be depleted from the cell preparation prior to implantation if they are to be used in conjunction with the induced membrane technique and bone graft substitutes, which have a very high surface/volume ratio.

## Figures and Tables

**Table 1:** Groups of different material and cell combinations and number of animals assigned in each group.

Material	Histology	Radiology/Biomechanical Testing
syngenic cancellous bone (SCB)	*n* = 5	*n* = 8
DBM granules (GDBM)	*n* = 5	*n* = 8
DBM granules +BMC (GDBM + BMC)	*n* = 5	*n* = 8
fibrous demineralized bone matrix (f-DBM)	*n* = 5	*n* = 8
fibrous demineralized bone matrix + BMC (f-DBM + BMC)	*n* = 5	*n* = 8

**Table 2:** Calculated bone healing scores in the different groups.

Material	Bone Healing Score
syngenic cancellous bone (SCB)	22
DBM granules (GDBM)	18
DBM granules + BMC (GDBM + BMC)	19
fibrous demineralized bone matrix (f-DBM)	20
fibrous demineralized bone matrix + BMC (f-DBM + BMC)	19

**Table 3:** Bone volume/total volume in the experimental groups. Mean values and standard deviation in % is shown.

Material	Bone Volume/Total Volume (BV/TV)
syngenic cancellous bone (SCB)	0.78 (±4.4%)
DBM granules (GDBM)	0.61 (±4.2%)
DBM granules + BMC (GDBM + BMC)	0.73 (±5.1%)
fibrous demineralized bone matrix (f-DBM)	0.64 (±6.1%)
fibrous demineralized bone matrix + BMC (f-DBM + BMC)	0.62 (±4.1%)

## Data Availability

The data presented in this study are available on request from the corresponding author.
